# The Future of Health Is Self-Production and Co-Creation Based on Apomediative Decision Support

**DOI:** 10.3390/medsci6030066

**Published:** 2018-08-22

**Authors:** Jack Dowie, Mette Kjer Kaltoft

**Affiliations:** 1 London School of Hygiene and Tropical Medicine, 15–17 Tavistock Place, London WC1H 9SH, UK; 2 University of Southern Denmark, J.B. Winsløws Vej 9B, 5000 Odense C, Denmark; mkaltoft@health.sdu.dk; 3 Odense University Hospital Svendborg, Valdemarsgade 53, 5700 Svendborg, Denmark

**Keywords:** medical futurists, paradigm change, disintermediation, apomediation, intermediation, patient preferences, decision support tool, decision aid, Multi-Criteria Decision Analysis

## Abstract

Cultural changes are needed in medicine if the benefits of technological advances are to benefit healthcare users. The *Digital Health Manifesto* of ‘medical futurist’ doctor Bertalan Meskó and ‘e-patient’ Dave deBronkart, *The Patient Will See You Now* by Eric Topol and *The Patient as CEO* by Robin Farmanfarmaian, are among the proliferating warnings of the approaching paradigm shift in medicine, resulting, above all, from technological advances that gives users independent access to exponentially increasing amounts of information about themselves. We question their messages only in suggesting they do not sufficiently shift the focus from ‘patient’ to ‘person’ and consequently fail to recognise the need for the credible, efficient, ethical and independent decision support that can ensure the ‘democratisation of knowledge’ is person empowering, not overpowering. Such decision support can ensure the ‘democratisation of decision,’ leading to higher quality decisions and fully-informed and preference-based consent to health provider actions. The coming paradigm will therefore be characterised by *apomediative* (‘direct-to-consumer’) decision support tools, engaged with by the person in the community to help them make health production decisions for themselves (including whether to consult a healthcare professional or provider), as well as intermediative (‘direct-from-clinician’) tools, delivered by a health professional in a ‘shared decision making’ or ‘co-creation of health’ process. This vision paper elaborates on the implementation of these preference-sensitive decision support tools through the technique of Multi-Criteria Decision Analysis.

## 1. Background: The Future and the Futurists

In this vision paper we take as given that the paradigm change in healthcare envisaged by ‘medical futurists’ such as doctors Eric Topol [1] and Bertalan Meskó [2] and patients Robin Farmanfarmaian [3] and Dave deBronkart [4], will happen. We fully endorse their broad diagnoses, prognoses and ‘therapies,’ except in the key respect that is the focus of this paper. There is a crucial omission in all their analyses, arising from their primary focus on *medicine* rather than healthcare and on patients rather than persons. They largely overlook the huge cognitive demands on the *person*, now able to access exponentially increasing amounts of information, who wishes to do so without resort to a health professional, since this is possible only as patient. The person qua person has little or no support, independent of health professionals, in entering the accessed information—possibly interesting but decisionally useless as such—into a coherent and appropriate decision framework. Our authors do not identify the ‘democratisation of decision’ as a necessary condition for the announced ‘democratisation of knowledge’ to realise its potential in health. Hence, they fail to consider person-focused, preference-sensitive decision support tools as essential in their digital future.

To justify these claims requires quoting at some length. We focus initially and mainly on the *Digital Health Manifesto* of Meskò and deBronkart [4].

The doctor-patient relationship in healthcare is on the verge of a paradigm shift… For centuries, the dominant paradigm has been that patients do not and cannot know what needs to be done (or more precisely cannot know anything worth knowing), yet in story after story, patients today are bringing real value to their own cases, thus “violating the paradigm-induced expectations that govern” the culture of medicine.

Thomas Kuhn wrote that when too many anomalies accumulate, a field goes into crisis until a new paradigm is developed and accepted. We assert that this crisis stage has arrived [in healthcare] but as often happens, the causes of the anomalies are poorly understood, which leads to confusion and confused reporting. No new paradigm can arise, letting the field advance, while confusion reigns.

Despite popular belief, disruptive technologies cannot bring change on their own. Social media, artificial intelligence, robotics, 3D printing, virtual or augmented reality and health sensors have no effect whatsoever without a change in stakeholders' attitudes and the structure of the system.

Another dimension arriving but not always welcome, is the reality that patients are gaining increasing data at home, continuing the time-honoured trends of home thermometers, home pregnancy tests, insulin test strips, CGMs and so forth. We can barely imagine the next step in which preliminary diagnoses and prescriptions may be possible. Virtual reality or augmented reality could bring doctors to patients’ homes (as telemedicine already does), which will be wonderful for the elderly or remote communities.

Very soon, the old paradigm “medicine is hard; people without medical training have nothing to offer” will be obsolete but we must work actively to change it among both patients and clinicians: it prevents most patients from speaking up, asking questions, telling their doctors their opinions, feelings and experiences—and even if they do, it prevents most doctors from recognizing the possible value and listening.

As long as the culture of medicine resists doing things differently, progress will be impeded. We must not construe shortfalls as the fault of technology if the culture hasn’t changed.

In summary, the Manifesto envisages

‘A future where old hierarchies tumble down, the paternalistic patient-doctor relationship is no longer needed and disruptive technologies enable the democratization of care by democratizing knowledge. A future where all these are in place due to cultural transformation facilitated by disruptive technologies. In short, a future where digital health has fulfilled its potential.’

The Manifesto suggests the move from ‘Traditional Medicine’ to ‘Modern Medicine’ and ‘Digital e-Health’ involves six fundamental changes:

- Point of Care: from Healthcare facilities to the Patient
- Reference Point: from Population to Individual
- Organising Principle: from Hierarchy to Partnership
- Data Ownership: from Institution to Patient
- Physicians Role: from Authority to Guide
- Knowledge Base: from Ivory Tower of Knowledge to Social Media and Crowdsourcing

All six of these fundamental shifts are convincingly described, analysed and justified in the *Manifesto* and an associated paper with Meskó as lead author [2]. Most, if not all, of the shifts are relatively uncontroversial in broad and abstract discussions of principle but movements to implement them in practice remain rare and hotly debated. The walking currently lags far behind the talking.

As self-proclaimed futurists Meskò and deBronkart are in excellent company. From the numerous candidates for inclusion, we select two: Eric Topol and Robin Farmanfarmaian.

Cardiologist and futurist Eric Topol in *The Patient Will See You Now: The Future of Medicine is on Your Hands* [1] argues that the smartphone is ‘the Gutenberg of medicine.’ As summarised by an anonymous reviewer:

Much as the printing press took learning out of the hands of a priestly class, the mobile internet is doing the same for medicine, giving us unprecedented control over our healthcare. With smartphones in hand, we are no longer beholden to an impersonal and paternalistic system in which ‘doctor knows best.’ Medicine has been digitized, now it will be democratized. Computers will replace physicians for many diagnostic tasks, citizen science will give rise to citizen medicine and enormous data sets will give us new means to attack conditions that have long been incurable. Massive, open, online medicine, where diagnostics are done by Facebook-like comparisons of medical profiles, will enable real-time, real-world research on massive populations. There's no doubt the path forward will be complicated: the medical establishment will resist these changes and digitized medicine inevitably raises serious issues surrounding privacy. Nevertheless, the result, better, cheaper and more human health care, will be worth it.

[5]

And, elsewhere

We are entering the age of “Do It Yourself” health care, smart phone care, retail care and cloud-based care. These changes are as disruptive to medicine as eCommerce and Megastores have been to retail.

[6]

Robin Farmanfarmaian, making the case for ‘*The Patient as CEO*’—and reaching a much wider public audience through the book that documents her own extraordinary patient journey [3]—sees us as

… on the cusp of a healthcare revolution. From wearable sensors, to improved point-of-care diagnostics to artificial intelligence and robotics, there are breakthroughs in biomedical technology on an almost daily basis which are set to fundamentally change the way that patients interact with their healthcare providers.

Farmanfarmaian aims to ‘show just how rapidly medicine is changing, empowering and enabling the patient to be a key decision maker’ [3] (p. 23). Patients will no longer be ‘afterthoughts’ or ‘sitting on the sidelines,’ but at the centre of ‘*patient-driven*’ rather than merely ‘*patient-focused*’ or ‘*patient-centred*’ medicine. Instead of being outside the inner circle that makes medical decisions, they will be dead-centre and they, and/or their carers, will be the ‘CEOs’ of their own healthcare team.

The explicit mention of carers provides an opportunity to emphasise that the presentation of the future visions in individual terms is not intended to deny or diminish the importance of the role of families and significant others in healthcare. This importance will vary from culture to culture and subculture to subculture and the reader is asked to accept, in what follows, that the concept of ‘person’ should be widened as appropriate to the context, for example to embrace the much greater and more active role of the family in health decision making in many—specifically non-Western—cultures.

While we can endorse the views of all these authors on how the arrival of active and passive technologies will transform the person’s life both inside their ‘smart home’ and outside it, they have one major shortcoming.

## 2. What’s Missing in All These Future Analyses?

This major shortcoming is ultimately derived from the focus on ‘patient’ rather than ‘person’ and the failure to follow through the implications of this key ontological distinction. The person-patient contrast is not ‘merely semantic’—to misuse the latter term in the common way—because the person *qua* person, not *qua* patient, must be at the heart of any coming paradigm change if it is to have real meaning and impact. While it is now common in the mental health setting to refer to persons rather than patients, this usage is only slowly diffusing beyond that context and in this respect our futurists are still in the present.

Meskó and deBronkart provide numerous indications of the ways in which *persons* will—or could—take control of and *self-produce* their own health. However, the fundamental assumption throughout the *Manifesto* is that healthcare professionals and providers will always be heavily involved in the health decision making process, albeit as ‘guide’ rather than ‘authority.’ ‘Co-creation’ of health by patient *and* medical professional will be the norm and the future of healthcare is almost completely equated with the future of medicine. (There is no suggestion, however, that they underestimate the effect of wider social determinants on the health of individuals and populations, whether ‘health’ and ‘healthcare’ are widely or narrowly defined).

Given this assumption, they are rightly concerned that persons arriving in the clinic to co-create their health, empowered and equipped by the outputs of new home-based technologies, will pose major challenges to healthcare professionals and organisations. Current training curricula and practice arrangements, as well as attitudes, reflect a culture that has not yet acknowledged the inevitably disruptive effects of citizen-empowering technological change. That *technological* changes mandate *cultural* changes is accordingly the central message of the *Manifesto*. Attempts to absorb these innovative technologies into the current culture—as just new ways to do old things—are doomed to fail and the blame will be improperly placed on the technology not fitting into the *status quo* culture, rather than the culture not changing to enable the benefits of the technology to be realised.

Susannah Fox, Chief Technology Officer at the U.S. Department of Health & Human Services acknowledges it is a little odd that her background is in anthropology, not technology. But, she says:

We’re living through this time right now where technology is a Trojan Horse for change. We say technology but we mean innovation. We say interoperability and open data but we mean culture change. And this is why the HHS CTO is an anthropologist. I know about culture change. I know how difficult it is for everyone involved. (http://www.mobihealthnews.com/content/hhs-cto-technology-healthcare-trojan-horse-culture-change)

However, in calling for cultural change, the *Manifesto* authors underestimate the massive material interests embedded in the present paradigm, in the form of personal and collective capital, both financial and professional. These material interests underpin the resistance to change which most often manifests itself in the broad claim that the suggested changes, while appealing *in principle*, will not be in the true interests of *our patients in practice*.

The new paradigm must therefore go even further than the disruptive future envisaged by Meskó and deBronkart, certainly beyond changes in encounter ‘communication’ and ‘shared decision making’ practices, even where these are substantive and not superficial. It must involve the creation of genuinely independent resource bases for person-driven health production and co-creation, accessible and delivered ‘direct-to-consumer/user/person/citizen.’

Following Eysenbach [7], we refer to these resources as ‘apomediative,’ because they ‘stand by’ the person but ‘away from’ (apo-) and independent of the provider of the good or service in question. Familiar examples of apomediative services are product and service comparison websites, such as ‘Which’ in the UK and ‘Taenk’ in Denmark. Apomediation is distinguished from ‘intermediation,’ where the provider decides what to tell the person on the basis of their perceived needs and requests and their own ability to deliver on options. Intermediation is therefore not provider-independent and options may be censored or filtered on the basis of the provider’s beliefs, values, or interests.’ Without being a requirement to access the product or service, apomediative resources and tools seek to help the person avoid such censoring and filtering and provide relevant and high quality independent guidance. In the other direction, apomediation is also distinguished from ‘disintermediation,’ where the person attempts to find what they want, or think they want, without any help, for example by doing an internet search.

The apomediative resources must include decision support, not just information support. Information alone cannot make a decision and information support is not decision support. Decision support requires showing how any information can be incorporated explicitly into a decision framework, making its impact observable and explorable.

In the light of this, the most significant aspect of the *Manifesto* is that two key words never appear: ‘decision’ and preference.’ While there are occasional *implicit* references to both, their absence reflects the central omission from the *Manifesto*, which is any mention of *personalised preference-sensitive decision support tools, engaged with by the person in the community*. Such tools are essential to provide the decision support based on the rapidly expanding range of self-sourcible inputs, including information obtainable from personal electronic health records. These tools will not only equip the person with increased competency in the self-production of their health but also enhance their ability to arrive at any subsequent co-creation encounter empowered *decisionally*, not merely *informationally*.

Farmanfarmaian and Topol, in documenting the contribution of new technologies to *information generation and processing*, fail to remedy this omission in the *Manifesto* by recognising and highlighting what the new digital technologies have to offer to *decision making*, the end use of information generation and processing. The absence of serious attention to preferences is noteworthy in their works also.

Repeatedly emphasising that ‘knowledge is power,’ there is no explicit recognition in Farmanfarmaian’s work that decisions require value-based preference judgements, as well as the knowledge and information which need to be integrated with them in decision making. Preferences are equally absent in Topol. While he may not himself have been responsible for the comprehensive 14-page index to his book, it is notable in not containing either the words ‘decision’ or ‘preference/s,’ let alone ‘decision aid’ or ‘decision support.’

In summary, in contrast to these fellow futurists, we argue that preference-sensitive, analytical decision support tools are essential if the person is to cope with and benefit from, the outputs of the new digital technologies—determining which of their informational outputs will be relevant inputs, as well as integrating them with their personal preferences in decision making.

Knowledge is only potential power: it empowers only when it is fitted into a coherent decision-making process. The ‘democratisation of decision’ is essential to yield the health and wider benefits of the ‘democratisation of knowledge.’ This was recognised by WHO in its 1998 endorsement of empowerment as the process by which people gain greater control over decisions affecting their health and the Aarhus Convention adopted three years later made public participation in decision making in environmental matters a human right issue (http://www.unece.org/env/pp/treatytext.html).

Apomediative personalised decision support tools built within Multi-Criteria Decision Analysis are the vehicle for this ultimate democratisation. This paper elaborates on the implementation of these preference-sensitive decision support tools through the technique of Multi-Criteria Decision Analysis.

## 3. Decisional Relationships and Apomediation

**Figure 1:**
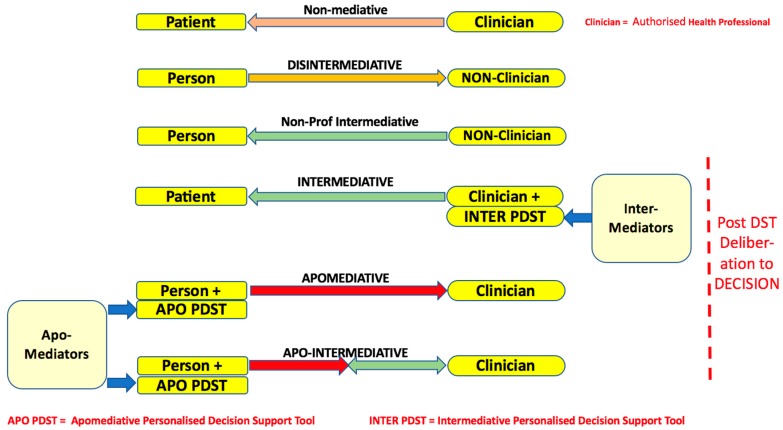
Taxonomy of alternative types of disintermediation, apomediation and intermediation.

The concepts of ‘apomediative’ and ‘intermediative’ decision support, based on the ideas of Gunter Eysenbach, provide the most helpful framework for addressing the main issues arising in the future roles and relationships of persons and healthcare professionals [7,8]. Figure 1 sets out the main possibilities for the relationship and types of decision support diagrammatically.

We now elaborate on each element of the taxonomy, starting from the top:

### 3.1. Non-Mediative Decision Support

The person accepts to be treated as a patient by a health professional, without any use of a decision support tool and to receive no explicit *decision* support other than that provided by the clinician in a standard consultation—which might be zero in ‘paternalistic’ medicine. The decision is taken for them, maybe with their complete agreement.

### 3.2. Disintermediative Decision Support

For some reason, perhaps dissatisfaction with non-mediative non-support as patient, the *person* bypasses health care professionals and providers and anonymously consults health-related resources—mainly information, possibly also some sort of ‘decision support tool,’ though this will not be one produced by an authorised healthcare professional organisation, or by an independent health decision analysis institute.

### 3.3. Non-Professional Intermediative Decision Support

The *person* enrols voluntarily for health-related support—mainly information but possibly including also a ‘decision support tool.’ Again, these will not be ones produced by an authorised healthcare professional organisation, or by an independent health decision analysis institute.

### 3.4. Intermediative Decision Support

The person accepts to be treated as a patient by a clinician who directs the encounter using an Intermediative Personalised Decision Support Tool (INTER PDST). Healthwise Decision Aids (https://www.healthwise.org) and Option Grids are examples. Some of these INTER PDSTs are publicly available, for example, Mayo Cards (http://centerforinnovation.mayo.edu/decision-aids) but many are available only through a healthcare provider on a commercial basis. Option Grids have recently moved from public access to access only via providers (https://health.ebsco.com/products/option-grid).

It is characteristic of most INTER PDSTs that they do not produce an opinion as to the best option or options and are designed and delivered with the aim of helping the patient to ‘make up their mind’ by whatever process they will use to do this.

### 3.5. Pure Apomediative Decision Support

An apomediative Personalised Decision Support Tool (APO PDST) assists the person in the community to

1. Make an informed and preference-based decision on whether to consult a Health Professional or provider, based on the opinion emerging from the tool
2. Prepare themselves for a professional consultation which they intend to arrange, or have already arranged, thereby coming to it empowered with the opinion generated by the tool. At arrival, there are four possibilities:
  (i) Pure apomediative route 1: the consultation follows the standard deliberative reasoning form and proceeds to a decision with little or no *explicit* reference to the tool by the person
  (ii) Pure apomediative route 2: the person requests that the clinician observes the output from the tool but does not ask them to engage with it online
  (iii) Pure apomediative route 3: the person requests that the apomediative tool is accessed as in-clinic apomediation, the clinician providing only ‘technical’ assistance in this engagement, not intermediative support
  (iv) Blended apomediative-intermediative route (see Section 3.7): the person requests that the apomediative tool is opened and engaged with jointly by both parties in the consultation, transforming it into a two-way apomediative -intermediative tool

A key characteristic of an APO PDST is that it is publicly accessible to any person in the community, whether or not they are currently labelled a ‘patient’ in the relevant area of healthcare. It is a ‘direct-to-consumer/user/citizen/person’ resource. If a person wishes to access the tool through an encrypted patient portal, this is possible if a relevant app exists that provides secure access to it.

It goes without saying that individuals will differ in whether and the extent to which, they wish to engage with home-based decision support in the form of APO PDSTs. That is why their informed consent to engage needs to be sought at the outset. This is particularly important because the simple experiencing of apomediative support is likely to have a value-clarifying, perhaps even value-constructing, effect. This reflective process, always raising the possibility of preference change, is likely to continue in any subsequent clinical encounter with a health professional.

### 3.6. Multi-Criteria Decision Analysis-Based Apomediative Decision Support

If Apomediative Personalised Decision Support Tools (APO PDSTs) are to meet the twin goals of preference-sensitive decisions and an informed and preference-based consent, we argue that the strongest analytical basis is provided by value-based, compensatory Multi-Criteria Decision Analysis (MCDA). (Other versions of MCDA which are either ranking-based or non-compensatory fail key requirements for preference-sensitive decision support). Intermediative aids (INTER PDSTs) will usually be multi-criterial but if they are not built in a transparent and theoretically-grounded technique such as MCDA, they will struggle to meet these twin requirements.

The essence of an APO PDST is that all the *personalising* inputs must be self-sourcible in the community, including information from the person’s Electronic Health Record (EHR) if it is accessible. The default evidence- and expertise-based ratings come pre-installed in the tool, along with the criteria and options.

**Figure 2:**
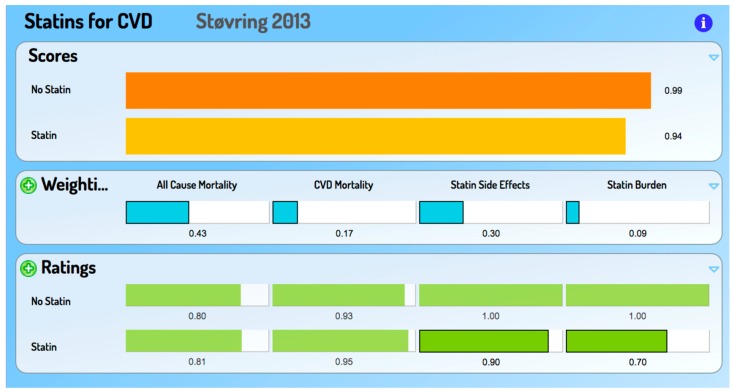
Final screen capture for Statin decision, reflecting pre-entered and supplied Ratings and Weightings.

The brief illustration of an APO PDST provided here is for the statin decision: Should I go, or not go, to my general practitioner to discuss taking statins? To engage with the tool, go to https://goo.gl/H7P51r. Figure 2 is an illustrative screen capture for a 60-year-old male smoker with a systolic blood pressure of 160 and a total cholesterol of 5.

The APO PDST involves the person:

- completing an online instrument to obtain an estimate of their personalised risks of All-Cause and Cardiovascular Mortality in the next ten years
- self-assessing their blood pressure and total cholesterol level, which are the two inputs required, along with age, sex and smoking status, to complete the EuroSCORE-based instrument
- self-rating the treatment burden of statins (in respect of burden ratings there may also be inputs from significant others, such as family and other carers, or caregivers if in a cared-for facility)
- assigning relative importance weights to the four criteria (10 year mortalities, statin side effects and statin burden).

This PDST includes All-Cause Mortality as well as CVD mortality, because the authors of the underlying paper [9] rightly stress that exclusive use of a condition-specific cause of mortality (such as CVD), gives the misleading impression that the person is immortal if they do not die from that condition. This paper was chosen partly because it includes this key criterion. However, any paper which reports the effect of alternative options on multiple criteria can be the basis of such an MCDA-based PDST, making these tools excellent vehicles for the translation of research findings into ‘bedside’ decision making. Numerous examples of such translation can be found at cafeannalisa.org.uk. We have shown elsewhere how uncertainty can be introduced into an MCDA-based PDST [10].

The ‘opinion’ from an APO PDST is never to be regarded as a *medical* opinion. However, being based on data well beyond that held by any individual health professional, using multiple criteria and being preference-sensitive, it could potentially be superior to a medical opinion for the person concerned.

### 3.7. Apomediative-Intermediative Decision Support

As suggested earlier, the person who has engaged with the APO PDST in the community may request that the health professional opens it in the consultation, in order that it can be used in a modified version of intermediation. This use is very much facilitated by the fact that it is already populated with the inputs from the person, that is, their criterion Weightings and their performance Ratings for the criteria where they are the expert, *par excellence* Treatment Burden.

In such blended ‘apo-intermediation,’ however, the clinician agrees to engage with the person in a way that is different—less controlling—from that which characterises the pure intermediative mode. This is symbolized in Figure 1 by the two-way intermediation arrow, compared with the one-way arrow in pure intermediation. For example, the clinicians will now need to be prepared to discuss options that are in the APO PDST that has been re-opened but may have been censored in the equivalent pure INTER PDST.

The professional is able to change a Rating in the opened APO-INTER PDST, where they believe they have a superior, more personalised, knowledge to that reflected in the APO tool. (This change is recorded by the program in a weblog). They may delete options on the basis of contraindications not known to the person but should not do so on the grounds of local restrictions or guidelines. (In contrast, they may and will often be doing this in delivering an INTER PDST). They can also add options but only on condition that they supply their personal performance Ratings for this option on *all the criteria*, except Respondent-rated criteria such as Treatment Burden. It cannot be emphasised too strongly that each and every Rating cell in the PDST requires a Best Estimate Available Now (BEAN).

Whether the tool is used in pure Apomediative or Apo-Inter form, it is important that engagement with it is not interrupted by any wider discussion of the decision that will be taken in the deliberative phase that is assumed to follow engagement with the tool (see far right of Figure 1). The validity of the opinion of the tool requires that it be delivered free from contamination. Ratings adjustment by the professional in Apo-Inter mode is *not* contamination. Changes of Weightings prompted by the autonomous person’s continuing reflection on and evolution of, their personal values and preferences—begun in the apomediative phase—are to be expected and encouraged. However, changes of Weightings prompted solely by the professional’s insertion of a ‘better’ set for this person, *are* contamination. Clinicians find this sort of interference in decision aid delivery hard to resist, even when they have committed to avoiding it [11,12].

The value of the PDST does not depend on the relationship of the opinion of the tool—post professional adjustment, if any—and the final decision. The opinion of the tool is defined as the complete set of option Scores, not just the ‘winner.’ The latter can only become a *medical* opinion if the clinician agrees with it.

## 4. The Future of ‘Shared Decision Making’

Where does the concept of ‘Shared Decision Making’ (SDM) fit into this envisioned future? Where the APO DST is used to decide whether or not to consult a healthcare professional and the decision is not to do so, the question of SDM or ‘co-creation’ of health does not arise. Where a pure INTER PDST is used, the precise nature and extent of SDM is indeterminate but is heavily loaded in the clinician’s favour in all respects. Where an APO PDST is used correctly in either Apomediative or Apo-Intermediative mode, there is necessarily extensive *role-specific sharing* in the *process* of decision-making. The Weightings and Treatment Burden Ratings are those of the person, while the Ratings are the Evidence- and/or Expert-based BEANs, possibly adjusted by the delivering professional on the basis of their local expertise or greater information about the particular person. (Note that this extra and valid information may change the BEANs without ‘flipping the DST opinion.’ This will especially be the case if the changes apply to a criterion that is lowly-weighted by the person).

A deliberative phase is assumed to follow delivery of the tool and the extent to which this deals fairly and transparently with the opinion of the PDST—either pure APO or APO-INTER—is a matter for the decision dyad. We stress that *the decision* is the person’s alone and in no way can be *shared*, even if *mutually agreed*. Aspects of the decision-making *process* can be shared in the deliberative phase and we believe that the person will be significantly empowered by the PDST in this final phase and this should positively affect the score on any Shared Decision-Making metric. More important, they can be expected to rate the eventual decision as of higher quality, using an appropriate dually-personalised formative instrument such as MyDecisionQuality (MDQ) [13].

The Achilles heel of conventional clinical medicine lies in its failure to separate ‘evidence’ from ‘values’ in an explicitly analytical and transparent way, a necessary condition for preference-sensitive person-centred care in a population with heterogeneous preferences. The same failure characterises the conventional ‘evidence base’ and guidelines, both of which seek to provide general conclusions and recommendation about *options*, inevitably based on average patients [14]. Almost universally applauded within the current paradigm, BMJ Rapid Reviews (https://www.bmj.com/rapid-recommendations) could be seen less positively as an attempt to avoid genuine person-driven preference-sensitive decision support at the point of care [15]. In the face of a ‘strong recommendation’ from such a source, it is unlikely a fully-informed and preference-based consent can be given by the person, because their personal preferences will neither be elicited formally, nor synthesised with the best available evidence/estimates for all options (established separately) in an explicit and transparent way.

## 5. Who Will Be the Apomediators?

In the coming ‘patient’-free paradigm, it will become evident that (a) Healthcare Analysis is not Healthcare and (b) a Healthcare Decision Analysis Professional is not a Healthcare Professional. And vice versa, in both cases.

The Healthcare Decision Analysis Professional is the appropriate person to perform the role of apomediator, since they are independent of the Healthcare Professional. The latter has increased information about the individual person, which *may* add to decision quality post apomediation but this advantage can easily be outweighed by material interests, professional obligations, organisational responsibilities and personal values, all of which threaten the delivery of genuine person-centred decision support.

Apomediative tools must therefore be provided through an organisation or institute that can offer, across the entire healthcare context, authoritative APO PDSTs, equivalent to that provided by *Which*, *Taenk* and *Choice* for other consumer goods and services. They will, however, offer analytical possibilities that go beyond those of such consumer organisations, to incorporate online interactive personalised preference-sensitive evaluations. The institute must be completely independent of pharma (http://medicalconsumerism.blogspot.co.uk), governments, professional guidelines and health service providers, since all have conflicts arising from pursuing their financial, political, organisational and/or professional interests and objectives. These conflicts go well beyond the conventionally reported ones—patchily reported according to the patient decision aid survey by Elwyn, et al. [16]—and extend to the deeper and long-standing concerns of critics such as Ivan Illich and George Bernard Shaw [17,18,19]. In instinctively opposing the creation of independent institutions of the required sort, health professionals and organisations would be well-advised to play the ‘trust card’ with great caution, because of the increasing public awareness of the conflicts and biases involved in the status quo.

A vital role for clinicians remains in the future but it will not be the type of partnership with patients envisaged by Shared Decision-Making advocates, including Meskó and deBronkart, Farmanfarmaian and Topol. The future of healthcare will be person-driven not provider-driven. The informational asymmetry underlying the principal-agent relationship of the current paradigm has disappeared with the ‘democratisation of knowledge.’ But this will be a superficial and unsustainable advance until it is followed by the ‘democratisation of decision.’ Note that it is democratisation of *decision* not democratisation of decision *making*. While the decision-making process can be shared, the decision itself cannot. The decision can be mutually agreed but it can be owned only by the person, or their non-professional proxy.

Apomediator’s competencies are in analysis, not care. They will leave the care to those with the *competency to deliver care*, which is completely separate from *competency to decide what care (testing and/or treatment) is in the best interest of the person*. Reluctance to accept this distinction between *analysis* and *care* is a sign of adherence to the current medical culture which fails to differentiate them. This reluctance frequently morphs into biased characterisations of transparent, quantitative decision analytical processes as ‘reductionist,’ ‘dehumanizing’ and ‘cookbook.’ Coming from people who have zero acquaintance with them, they can be expected to disappear with the paradigm that harbours such prejudices.

Decision makers (e.g., medical doctors) will in future need to show they can improve on the opinion of an APO PDST, not simply assume and assert that they can. The results from the most relevant studies shows that this will be extremely difficult, as the work of Paul Meehl established long ago in the clinical versus actuarial controversy [20]:

When you are pushing 90 investigations, predicting everything from the outcome of football matches to the diagnosis of liver disease and when you can hardly come up with half a dozen studies showing even a weak tendency in favour of the clinician, it is time to draw a practical conclusion, whatever theoretical differences may still be disputed… If I try to forecast something important about a college student, or a criminal, or a depressed patient by inefficient rather than efficient means, meanwhile charging this person or the taxpayer 10 times as much money as I would need to achieve greater predictive accuracy, that is not sound ethical practice. That it feels better, warmer and cuddlier to me as predictor is a shabby excuse indeed.

[20] (p. 374)

It is unlikely that health care professionals can be apomediators, wearing two hats. In order to become a formal part of the development of apomediative decision support tools for the person, health care professionals would have to give up their membership of the medical community, because of the conflicts of interest it creates: no loyalty or commitment, either personal or professional, to principles or to authorities, can be allowed to interfere with the independent generation of the apomediative product. In any case, most clinicians will lack and would need to acquire, the necessary decision analytic competence and sub-competencies in epidemiology and other relevant methodologies. These shortcomings and the exclusion of serious attention to analytical decision making in medical curricula, are not accidental. They reflect the un-evidence-based faith in intuitive reasoning, tacit knowledge and ‘reflective practice,’ none of which (despite their undoubted existence) will be able to survive, in their current form, in the *transparent* paradigm that is coming.

If clinicians choose to remain inside the conventional practice box, they will be (as Meskò and deBronkart warn) increasingly annoyed by their encounters, especially with APO PDST-empowered patients who are not willing to switch into intermediative mode. Their claims to ‘tacit knowledge’ will be treated with scepticism unless and until it can be externalized in a decision model. Tacit knowledge *is* knowledge but it can only be a contributor to a personalised decision support tool if it can improve the BEANs in an explicit Ratings matrix, which means it has to be ‘published’ and no longer be tacit.

The upside for medicine is that by devolving *decision* responsibility, physicians can rid themselves of the burden of choosing the ‘right’ therapy and bearing the consequences. Accepting their limited but important role in the co-creation of the health of the person, on the basis of apomediative decision support tools used apo-intermediatively (perhaps via tele-medicine), will constitute a win-win for both parties. Providing empathy and the human touch will be the essence of the future *care* provided by healthcare professionals. That is not something which an APO PDST can deliver, even though it should provide opt-ins and links to support of kinds that go beyond its focus on decision making. That must always remain its unremitting primary focus—and sole justification.

## 6. Is the Future Already Here? ‘Realistic Medicine’ in Scotland

Catherine Calderwood, on taking up the position of Chief Medical Officer, launched ‘Realistic Medicine’ as the way forward for medical care in Scotland. Her promotion of it as the way to better Shared Decision Making is now in its third year [21]. The objectives—and anticipated benefits—are clear from the sentiments expressed throughout her 3rd Annual Report.

Practicing Realistic Medicine requires care that is coproduced in partnership with the people receiving it—person-centred, holistic care… We must move away from the “Doctor knows best” culture and generate supportive environments where people truly feel comfortable asking questions about their care and can expect to get clear answers… A key part of shared decision making is honesty and realism about possible outcomes; recognising the benefits but also the risks and limitations of treatment in the context of the patients’ life and what matters most to them… People must be empowered to discuss their treatment fully with their healthcare providers including the possibility that a suggested treatment might come with side effects—or even negative outcomes. Everyone should feel able to ask their doctor why they have suggested a test, treatment or procedure and all decisions about a person’s care should be made together… We know that people want to be more involved in decisions about their care, yet they may not know what to ask, or feel that they don’t have “permission” to participate in the decision-making process… We know that patients express more satisfaction during consultations where they have been able to express their perspective and objectives—what matters most to them…. Not only are people more likely to have greater confidence in reaching decisions through this person-centred approach but there is evidence that adherence to treatment is improved as well as patients experiencing less regret about treatment choices.

However, it is clear from the extracts above that Realistic Medicine mainly involves correcting aspects of current practice that are ethically unacceptable in normal, let alone ‘person-centred,’ care. We also read:

While professionals will be used to engaging in discussions about informed consent, going a step further by asking about the person’s perspective, health beliefs and preferences is a highly skilled interaction.

The suggestion that informed consent can be discussed, even obtained, *without* asking about the person’s perspective, health beliefs and preferences, is worrying, even if the desired consent does not approach the ’perfected’ informed level advocated by Nelson and Clay [22].

So, it is clear that the future is not already here and will not be, even if the goals of Realistic Medicine are fully realised. Focusing on the basics of honest communication between clinician and patient on both information and preferences, Realistic Medicine might be seen as, at best, making the case for clinician-developed and delivered intermediative decision aids. It shows no awareness of the possibility of apomediative decision support tools, or the role they could play in empowering the person—the person who can come to the clinical encounter with a preliminary opinion that ensures they are well-equipped to participate in the conversation and give truly informed and preference based consent to any future action. The person will then drive the Shared Decision Making, including agreeing to segue into apo-intermediative mode.

## 7. The Person Is Also a Citizen

Seeking to avoid the fundamental conflict between person-centred care and policy-level decisions based on effectiveness and/or cost-effectiveness is as doomed as the hunt for the mythical Snark [23]. The person is also a citizen and, in a resource-constrained, publicly-funded system must simply endorse the principle of cost-effectiveness on ethical as well as efficiency grounds. Each person must count equally in person-centred care, so the opportunity cost of using resources on any one person must impact on clinical decisions, as well as the higher-level allocation ones.

This does *not* mean that it is ethical for the person to be treated as a means to achieve some policy end—to reduce the incidence of this, increase the uptake of that—even if it is in their *perceived* individual best interests. If the person’s ‘undesirable’ preferences reflect their socioeconomic and cultural contexts, which they may well do, it is these which need to be addressed directly, not worked-around by delivering public health via a clinical medicine that is not person-centred. A bottom-up approach of bringing the person’s decisions into the formulation of the policy goals, through preference-sensitive support that uses the best available estimates of *multi-criterial* effectiveness, is the way forward. MCDA-based decision support is as valuable at the group policy-level as at the individual decision one [24].

It is also important to stress that the individual can only self-produce their health and co-create it with healthcare professionals, within their biological givens, the wider environment, socioeconomic constraints and conditions in which they live and work. As the Meikirch model asserts

Health is a state of wellbeing emergent from conducive interactions between individuals’ potentials, life’s demands and social and environmental determinants… Health occurs when individuals use their biologically given and personally acquired potentials to manage the demands of life in a way that promotes well-being. This process continues throughout life and is embedded within related social and environmental determinants of health.

[25] (pp. 363, 369)

There are therefore grave dangers in overstating the extent to which health is determined through individual healthcare, rather than at the community and higher levels. Most of the recent advances in key health indicators (e.g., life expectancy) are attributable to sectors other than healthcare, so that health needs to be approached within a much wider and comprehensive policy portfolio. Equitable and cost-effective cross-sectoral public health policies are an essential complement to the present restricted vision of the future.

## 8. Conclusions: Back to the Futurists

Robin Farmanfamaian and e-patient Dave deBronkart are individuals who have drawn on their stressful experiences prior to diagnosis and during their continuing journey as patients. Their highlighting of ‘patient’ is fully explained by the painful facts of their life experiences. Berci Meskò is a healthcare professional who, via Webicina (http://webicina.com) has, for many years, curated evidence/literature from the entire range of professional and social media relevant to patients and health professionals in multiple languages. He is now using himself as a test-bed to explore all the wearables and other self-sourced inputs that can improve healthcare provision. Eric Topol is a world-renowned clinician, as well as innovator and communicator about the way in which new technologies require the ‘creative destruction’ of medicine as we know it [26].

As emphasised earlier, it is only in one respect that we seek to add value to their major contributions. Re-focusing on the person rather than the patient will heighten the awareness in all parties of the new analytical decision support competencies that are required, *by both persons and professionals*, to turn the ‘information explosion’ into more and better health. In our view, Multi-Criteria Decision Analysis is the best vehicle for moving into this future and, as a completely different technique from clinical reasoning, it encapsulates the need for *cultural change* in medicine and medical training that goes far beyond the greater IT literacy and computer competence it requires.
